# Predicting the Mass Adoption of eDoctor Apps During COVID-19 in China Using Hybrid SEM-Neural Network Analysis

**DOI:** 10.3389/fpubh.2022.889410

**Published:** 2022-04-28

**Authors:** Qing Yang, Abdullah Al Mamun, Naeem Hayat, Mohd Fairuz Md. Salleh, Anas A. Salameh, Zafir Khan Mohamed Makhbul

**Affiliations:** ^1^UCSI Graduate Business School, UCSI University, Kuala Lumpur, Malaysia; ^2^UKM-Graduate School of Business, Universiti Kebangsaan Malaysia, Bangi, Malaysia; ^3^Global Entrepreneurship Research and Innovation Centre, Universiti Malaysia Kelantan, Kota Bharu, Malaysia; ^4^College of Business Administration, Prince Sattam Bin Abdulaziz University, Al-Kharj, Saudi Arabia

**Keywords:** eDoctor apps, perceived compatibility, perceived usefulness, perceived technology accuracy, perceived privacy protection, artificial neural network, PLS-SEM

## Abstract

Technology plays an increasingly important role in our daily lives. The use of technology-based healthcare apps facilitates and empowers users to use such apps and saves the burden on the public healthcare system during COVID-19. Through technology-based healthcare apps, patients can be virtually connected to doctors for medical services. This study explored users' intention and adoption of eDoctor apps in relation to their health behaviors and healthcare technology attributes among Chinese adults. Cross-sectional data were collected through social media, resulting in a total of 961 valid responses for analysis. The hybrid analysis technique of partial least squares structural equation modeling (PLS-SEM) and artificial neural network (ANN) analysis was applied. The obtained results revealed the significant influence of eDoctor apps in terms of usefulness, compatibility, accuracy, and privacy on users' intention to use eDoctor apps. Intention and product value were also found to suggestively promote the adoption of eDoctor apps. This study offered practical recommendations for the suppliers and developers of eHealth apps to make every attempt of informing and building awareness to nurture users' intention and usage of healthcare technology. Users' weak health consciousness and motivation are notable barriers that restrict their intention and adoption of the apps. Mass adoption of eDoctor apps can also be achieved through the integration of the right technology features that build the product value and adoption of eDoctor apps. The limitations of the current study and recommendations for future research are presented at the end of this paper.

## Introduction

In the past decade, the application of smart computing has attracted a lot of attention, especially since various smart products and smart applications produced through smart computing can be connected through private networks and media in ubiquitous environments, thus increasing the convenience of human daily life and making people's daily lives easier and more efficient, such as healthcare systems and online healthcare platforms (1). The recent development of information technology has propelled more healthcare organizations to provide healthcare services through a virtual platform (2). For instance, e-health devices and wearable medical devices are introduced to provide patients and clients on-the-go healthcare services. With the timeliness of the Internet, users can regularly obtain health-related information through their electronic devices, anytime and anywhere, thereby supporting self-management of health and helping to alleviate various key challenges that offline hospitals are facing. The advent of e-health devices has provided many individuals and patients the needed regular healthcare services and medical consultations, especially those who do not intend to leave home due to their concerns of COVID-19 infection (3).

With the development of the Internet and its widespread use, technologies and software that bring convenience to the lives of the masses are being innovated, developed, and utilized one after another, such as online medical and healthcare platforms, especially during COVID-19, when many online medical platforms and personal health management applications were frequently used in China (3). Through technological innovation, more healthcare organizations consider using information technology and digital technologies to develop online platforms in order to connect and communicate with the public and patients, especially during COVID-19. The application of artificial intelligence has had a huge impact on smart healthcare systems, allowing people and patients to have access to health advice outside of hospitals, and to diagnose, prevent, and treat their own illnesses (mental or physical) through such smart healthcare systems and applications to facilitate timely treatment and health improvement (4). Due to the rapid growth of smartphones worldwide, software applications such as the available eDoctor apps are of great potential for patients to communicate with their doctors and obtain timely clinical diagnosis and treatment recommendations from their doctors or specialists (2, 5). It also solves some of the difficult problems encountered by patients with chronic diseases, such as difficulties of buying medications and obtaining regular medical consultations (6).

With the widespread use of the Internet and 5G networks in China, Internet healthcare platforms, eDoctor apps, and e-health products are now widely accepted and used by the Chinese public, with services such as online consultations, online care, and ongoing diagnosis and treatment for specific patients (chronic diseases) available (3). The majority of Chinese people who use eDoctor apps and related apps (such as the HUAWEI watch and portable blood glucose meter) choose to use eDoctor apps and related apps (such as the HUAWEI watch and portable blood glucose meter) to track and monitor their physical diseases, such as cardiovascular disease and hypertension, using data analysis from eDoctor apps (7). Chinese healthcare enterprises have begun to flourish and expand as a result of technology advancements and the aging of society.

Factors influencing the adoption of m-health products in different countries have been explored in numerous studies, but users' intention to use healthcare apps and perceptions of using healthcare apps have remained underexplored. In this regard, several prior studies argued the important roles of users' personal perceptions and consciousness of and motivation for personal health in terms of their intention to use e-physician apps. Meanwhile, prior studies noted the influence of health consciousness (HCS) and health motivation (HMO) on users' intention to use such apps (8–10). As HCS and HMO increase, users become more skeptical on the purposeful claims of the products, which subsequently influence their perceptions of the products (11), including perceived product value (PPV) and product technology. Most studies adopted technology acceptance model (TAM) and examined perceived credibility, perceived ease of use, and perceived usefulness (PUS) as factors that influence users' perceptions or intuitive thoughts toward technology-related products and services (12). However, there have been limited findings on the effects of other predictors like perceived compatibility (PCT), perceived critical mass (PCM), perceived privacy protection (PPP), and perceived technology accuracy (PTA) on users' perceptions of the technology or product itself.

Therefore, this study aimed to examine the effects of HCS, HMO, PCT, PCM, PUS, PTA, and PPP on users' intention to use eDoctor apps (ITU) and adoption of eDoctor apps (ADT), as well as the mediating effects of ITU on the hypothesized relationships and the moderating effect of PPV on the relationship between ITU and ADT. The current study focused on Chinese adults' inclination toward personal health, and health technology attributes harnessing the intention and adoption of health technologies like eDoctor apps.

The next section describes the theoretical basis of the current study and the development of hypotheses. The methodology adopted in this study is also described in the subsequent section. The obtained results are then discussed in the light of prior literature. The implications and limitations of this study are described at the end of this paper.

## Literature Review

### Theoretical Foundation

Several theoretical models have been developed to understand the acceptance and use of information systems. Focusing on the dilemma of selecting an appropriate theoretical model to understand the acceptance and use of information systems, the use of Unified Theory of Acceptance and Use of Technology (UTAUT) has been regarded as an appropriate theoretical model to assess the acceptance and use of technology (13). In order to choose an appropriate model that covers almost all factors that influence users' intention to use and adopt eDoctor apps, UTAUT was specifically used as a theoretical basis for the conceptual model presented in this study. Based on the existing literature, PCT, PCM, PUS, PTA, PPP, and PPV were identified as the main factors influencing users' intention to use and adopt eDoctor Apps. In order to develop a comprehensive model that measures usage in any setting, this study attempted to extend the UTAUT model with the most common and important factors discussed in eHealth-related literature. This study proposed that PCT, PCM, PUS, PTA, PPP, and PPV influence users' intention to use and adopt eDoctor apps.

### Development of Hypotheses

#### Health Consciousness and Health Motivation

HCS is an intrinsic personal preference or consumption characteristic that affects one's motivation for health behaviors and, in turn, health-related behaviors (14). HCS involves similar self-consciousness that motivates health vigilance and self-monitoring (15). Therefore, HCS and HMO are expected to motivate health behavioral compliance. HMO represents the willingness and interest of performing health-related behaviors (16). When it comes to the specific context of health-related behavioral change, the willingness and motivation to engage in health-related behaviors (i.e., the level of HCS) play an essential role in one's behavior. Several prior studies demonstrated the significant and positive influence of HMO on behavioral intention. This factor can determine one's perception of the values of health and safety. (17) considered HMO as a process that involves choices, competence needs, and self-determination of one's health (8). Therefore, this study examined HCS and HMO as factors that influence users' intention to use and adoption of eDoctor apps. The following hypotheses were proposed for testing:

H_1_: *HCS positively influences ITU*.
H_2_: *HMO positively influences ITU*.

#### Perceived Compatibility

PCT refers to the extent to which the experience is perceived to be consistent with one's existing values, beliefs, habits, and present and past experiences (18). Many prior studies demonstrated that, when users move from offline to online channels for shopping and consumption, the PCT between both channels clearly affects their purchase intention, which in turn affects their behavior (19). Recent studies have demonstrated that users' previous experience can predict their behavioral intention and adoption (20), implying the influence of users' perceptions of compatibility on their use of products or technology adoption (21). Based on the review of literature, this study proposed the following hypothesis:

H_3_: *PCT is positively related to ITU*.

#### Perceived Critical Mass

Rogers (22) defined critical mass as having enough people adopting an innovation, resulting in mass adoption rate and subsequently, sustain itself in development. Although it is difficult to calculate the actual threshold of critical mass for a particular technology, individuals may have their own perceptions and opinions about the threshold of critical mass for the technology. Studies have conceptualized such perception as PCM (23, 24) and noted its significant influence on users' decision to adopt a new technology (24, 25). Prior studies revealed the influence of PCM on the acceptance and use of technology, and many studies identified PCM as the key determinant of user acceptance of technology (23, 26). Strader et al. (25) defined PCM as a value-oriented concept that influences user acceptance of a new technology or product acceptance behavior. Thus, the following hypothesis was proposed for testing in this study:

H_4_: *PCM is positively related to ITU*.

#### Perceived Usefulness

Perceived usefulness has been shown to be the most critical factor in user acceptance of a new system or technology in the Technology Acceptance Model (TAM) introduced by Davis (27), and thus perceived usefulness (PUS) can determine an individual's acceptance of that system or technology. E-health literature in the last two recent years has revealed the development of higher perceptions on the usefulness of smartphones among many smartphone users, as most users perceive their health to be at risk to some extent due to COVID-19 (28). Leung and Chen (29) identified PUS as a significant predictor of user satisfaction and continues intention. Kim and Park (30) and Ambalov (31) also confirmed the positive and significant influence of technological innovation and information provision of smartphones on PUS and the influence of PUS on intention to use eDoctor apps. Therefore, the following hypothesis was proposed in this study:

H_5_: *PUS is positively related to ITU*.

#### Perceived Technology Accuracy

Alam et al. (32) claimed the direct influence of PTA and perceived trust on users' intention to use and adopt e-health products and other related services. Sharma and Sharma (33) reported the significant impact of technology accuracy on users' intention to use and adoption of technology. Reliability, authenticity, privacy, and security are the primary concerns of most users when they consider the adoption of e-related products (34). In many studies, PTA clearly influences the intention to adopt and actual of adopting m-Health products and services (35, 36). Based on the review of literature, the following hypothesis was proposed for testing:

H_6_: *PTA is positively related to ITU*.

#### Perceived Privacy Protection

The issue of user trust in electronic products has been widely discussed, extensively studied, and explained in different ways. User psychology can be seen as a psychological dependency relationship, and user trust is also often based on moral code and past commitment (37). Many previous studies examined users' perceptions of trust as a key factor to investigate the impact of perceived risk on intention to use (38). This suggests that privacy protection can effectively increase users' perceptions of trust and intention to use (38, 39). Raschke et al. (40) examined the impact of privacy beliefs on behavioral intention. The study revealed the significant impact of both privacy protection and privacy risk beliefs on behavioral intention and showed the negative relationship between privacy protection beliefs and privacy concerns. Based on the findings of previous studies, the following hypotheses were proposed for testing in this study:

H_7_: *PPP is positively related to ITU*.

#### Intention to Use eDoctor Apps

Intention to use technology is a central factor in both TAM (41) and UTAUT (42). Intention to use technology can also be used to predict users' actual use of technology. Such intention has been studied in theoretical studies related to both TRA and TPB; intention to use, which indicates one's willingness to try or make an effort to perform the said behavior, is the most influential predictor of behavior (43). Prior studies used intention to use as a dependent variable. In this study, ITU was used as one of the dependent variables to investigate users' perceived consciousness and motivation.

In most research frameworks, there is a correlation between users' intention to use and their actual behavior, that is, the presence of some factors that directly or indirectly influence their intention to use and consequently, actual actions (44). In the context of e-health products and services, many users may intend to use this type of e-service, but certain personal perceptual factors may influence their actual use of the product. As for the current study, the levels of consciousness and motivation regarding users' own health, as well as users' perceptions of the product itself (including PCT, PCM, PUS, PTA, and PPP), were postulated to influence users' ITU, which may translate into actual use of the product.

#### Adoption of eDoctor Apps

Intention to use is a central factor in TAM (41). Many empirical studies that adopted TAM confirmed the positive influence of consumers' perceptions of a product on their intention to use the product (45–47). However, technology adoption is also a noteworthy factor to explore. Therefore, this study examined both ITU and ADT as dependent variables to obtain better understanding on users' perceived consciousness and motivation. Earlier studies on usage behavior were primarily designed to explore the relationship of how users generate intention and how users engage in the actual behavior. These studies focused on the antecedents and consequences of users' behavioral intention and actual behavior. Previous studies did not concurrently examine the relationships of three key concepts: motivation, intention, and adoption. Thus, the current study examined users' ITU and ADT with respect to the combination of both TRA and UTAUT. In view of the above, the following hypothesis was tested:

H_8_: *ITU is positively related to ADT*.

#### Mediating Effects of ITU

Personal HCS and HMO are relevant in building inclination and adoption of health-related technologies. The technology-related aspects of compatibility, usefulness, technical accuracy, and privacy harness users' intention to adopt and actual adoption of health-related technologies. As a social phenomenon, technology adoption vastly depends on the mass adoption of technology by the community. Multiple perceptual factors nurture technology adoption through the formation of intention. Therefore, the current study proposed the mediating effects of ITU on the hypothesized relationships of personal health perceptions and technology-related attributes in relation to ADT:

H_9M_: *ITU mediates the relationships of HCS, HMO, PCT, OCM, PUS, PTA, and PPP with ADT*.

#### Moderating Effect of Perceived Product Value

Product value works as a form of perception derived from the value of money consumers pay for a product and the benefits derived from the product use (48). Consumer-level perception of products nurtures one's behavioral intention and subsequently, the adoption of the right products or services (49, 50). Users' inclination toward novel technologies shifts from intention to adoption based on the higher perception of product value (51). Chen (48) postulated that the perception of product value triggers one's intention toward the actual use of the technology. E-health services, as a form of technology, offer unique features and benefits, which may have contributed to the formation of PPV and suggestively instigated ADT (50). Thus, the following hypothesis was proposed for testing:

H_10_: *PPV moderates the relationship between ITU and ADT*.

**Figure 1:**
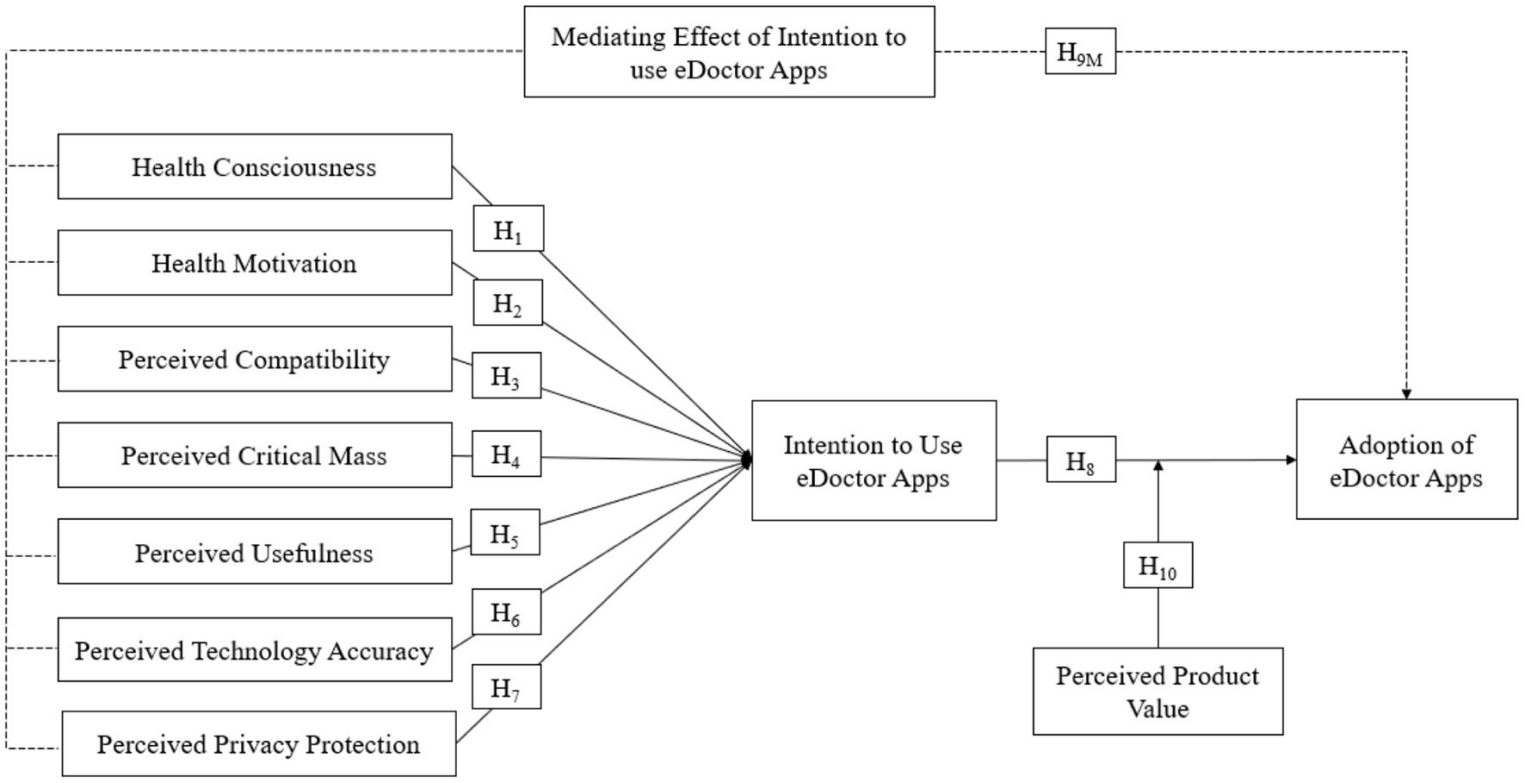
Research framework.

The research framework (shown in Figure 1) highlighted the hypothesized associations, such as the effect of health consciousness, health motivation, perceived compatibility, perceived critical mass, perceived usefulness, perceived technology accuracy, and perceived privacy protection on the intention and adoption of eDoctor apps, based on the discussion presented in the literature review section. The moderating influence of perceived product value was also underlined in this study.

## Methodology

### Data Collection

Quantitative analysis was used to test the correlation between variables. A cross-sectional study and a self-administered questionnaire were used for data collection. The target population of the current study involved Chinese adults, representing 65% of the total Chinese population. The determination of sample size was conducted using G^*^Power 3.1. With the effect size of 0.15, power of 0.95, and 10 predictors, the appropriate sample size required was 172 (52). However, Hair et al. (53) recommended using a sample size of at least 200 for PLS-SEM. The convenience sampling strategy was employed to obtain sufficient respondents for the questionnaire survey. The questionnaire was uploaded on WJX and dissemination *via* WeChat, and a few qualifying questions were used to filter the potential respondents. The online questionnaire was uploaded via a specific link: https://www.wjx.cn/vj/hFtJ7Zw.aspx. As a result, 961 valid responses were successfully obtained for the final analysis.

### Instrument

Overall, the developed questionnaire consists of two parts: Section A and Section B. Section A focuses on the demographic characteristics of respondents, such as gender, age, monthly income, education level, and current location. At the same time, respondents were required to indicate whether they have used eDoctor apps; this was expected to help the respondents to have a better understanding on the scope of this survey. Section B focuses on HCS (54), HMO (55, 56), PCT (57), PCM (57), PUS (57), PTA (58), PPP (7), PPV (59), ITU (7, 60), and ADT (61). For this study, all 46 measurement items were adapted from prior studies. A seven-point Likert scale, ranging from 1 (strongly disagree) to 7 (strongly agree), was used to evaluate the measurement results. The complete questionnaire is presented in Appendix 1.

### Common Method Bias

For this study, CMB was evaluated with the full collinearity test recommended by Kock (62). All latent constructs in this study regressed on a common created variable. Variance inflation factor (VIF) values for HCS (1.729), HMO (2.208), PCT (3.968), PCM (2.934), PUS (1.628), PTA (2.627), PPP (2.354), PPV (2.987), ITU (4.654), and ADT (3.155) were <5, suggesting the non-existence of collinearity in this study (63). Next, correlations of latent factors were evaluated to estimate the CMB issue. These correlations must be <0.90 to confirm the non-existence of the CMB issue (64). The correlations among these latent constructs did not exceed 0.90 (the correlation between ITU and ADT recorded the highest correlation value of 0.771). In other words, the CMB issue was not significant for the current study.

### Multivariate Normality

Checking multivariate normality is essential to select the appropriate data analysis. For this study, the multivariate normality was evaluated using the Web Power online tool (Source: https://webpower.psychstat.org/wiki/tools/index). The calculated Mardia's multivariate *p*-value revealed that the study data had non-normality issue, as the recorded *p*-values were below 0.05 (65). The study data demonstrated the issue of non-normality. With that, the current work study assumed partial least squares structural equation modeling (PLS-SEM).

### Data Analysis

This study performed PLS-SEM using the PLS software to assess the structural model. Since PLS-SEM only provides standard linear models (66), and considering the complex nature of social sciences, using linear models alone is not enough. Addressing this issue, the current study incorporated artificial neural network (ANN) analysis to complement the interpretation of non-linear relationships beyond PLS-SEM. Significant predictors identified in PLS-SEM served as input neurons for the ANN model. Finally, this study evaluated measurement invariance of composite models (MICOM) using a composite model. This involved three steps: (1) configural invariance; (2) compositional invariance; (3) the equality of composite mean values and variances. The extended composite detection method was used to evaluate both structural invariance and measurement invariance.

## Results

### Demographic Characteristics of Respondents

This study collected data from a total of 961 respondents. Table 1 presents the demographic profile of respondents. In particular, most of the respondents (55.8%) were female, and the remaining 44.2% were male. Besides that, 69.4% of the total respondents were between 20 and 30 years old, followed by the age groups of 31–40 years old (20%), 41–50 years old (5.3%), and 51–60 years old (4.3%). The other 1.0% of the total respondents were above 60 years old. Most importantly, only 29.1% of the total respondents reported to have experience in using healthcare monitoring devices.

**Table 1:** Demographic profile of respondents.

	***n***	**%**
**Gender**		
Male	425	44.2
Female	536	55.8
Total	961	100.0
**Education**		
Secondary school certificate	56	5.8
Diploma/technical certificate	134	13.9
Bachelor degree or equivalent	460	47.9
Master degree	261	27.2
Doctoral degree	50	5.2
Total	961	100.0
**Provinces**		
Beijing	91	9.5
Shanghai	87	9.1
Guangdong	45	4.7
Guangxi	44	4.6
Zhejiang	54	5.6
Shandong	61	6.3
Hunan	11	1.1
Jiangsu	114	11.9
Others	454	47.2
Total	91	9.5
**Age**		
20–30 years	667	69.4
31–40 years	192	20.0
41–50 years	51	5.3
51–60 years	41	4.3
Above 60 years	10	1.0
Total	961	100.0
**Monthly income**		
Below CNY 2500	292	30.4
CNY 2501–5000	210	21.9
CNY 5001–7500	168	17.5
CNY 7501–10,000	99	10.3
CNY 10,001–12,500	70	7.3
Above CNY 12,501	122	12.7
Total	961	100.0
**Do you use any healthcare monitoring device?**		
Yes	280	29.1
No	681	70.9
Total	961	100.0

*Source: Author's data analysis*.

### Validity and Reliability

When it comes to analyzing the outer measurement model, it is necessary to first verify the reliability and validity of the questionnaire (53). The consistency of the measurement structure of the scale is determined by reliability (67). Previous studies typically recommend using Cronbach's alpha, composite reliability, and Dijkstra-Hensele's rho to accurately determine internal consistency (68, 69). The results in Table 2 revealed that all values of Cronbach's alpha, composite reliability, and Dijkstra-Hensele's rho exceeded the threshold value of 0.7, indicating the reliability of the internal consistency of the questionnaire (69). Meanwhile, validity is divided into convergent validity and discriminant validity. Convergent validity in this study was identified by average variance extracted (AVE) and factor loadings. Referring to the results in Table 2, the recorded AVE value of each latent variable exceeded 0.5, indicating acceptable convergent validity of all latent variables (53).

**Table 2:** Reliability and validity.

**Variables**	**Items**	**Mean**	**Standard deviation**	**Cronbach's alpha**	**Dijkstra-Hensele's *rho***	**Composite reliability**	**Average variance extracted**	**Variance inflation factor**
HCS	5	5.697	1.143	0.903	0.911	0.928	0.721	1.701
HMO	5	5.643	1.093	0.914	0.922	0.936	0.745	2.189
PCT	5	5.319	1.213	0.960	0.960	0.969	0.861	3.830
PCM	5	4.972	1.419	0.948	0.949	0.960	0.828	2.731
PUS	5	5.129	1.213	0.951	0.953	0.962	0.837	1.563
PTA	5	5.173	1.211	0.954	0.955	0.965	0.846	2.125
PPP	5	5.316	1.166	0.932	0.935	0.949	0.787	2.032
PPV	5	5.182	1.202	0.958	0.959	0.968	0.857	2.357
ITU	5	5.244	1.210	0.966	0.966	0.974	0.880	2.357
ADT	1	5.084	1.302	1.000	1.000	1.000	1.000	

*HCS, Health consciousness; HMO, Health motivation; PCT, Perceived compatibility; PCM, Perceived critical mass; PUS, Perceived usefulness; PTA, Perceived technology accuracy; PPP, Perceived privacy protection; PPV, Perceived product value; ITU, Intention to use eDoctor Apps; ADT, Adoption of eDoctor Apps*.

*Source: Author's data analysis*.

Discriminant validity is typically determined based on Fornell-Larcker criterion and heterotrait-monotrait ratio of correlations (HTMT). Fornell-Larcker criterion values in Table 3 shows the square root value of AVE of each latent variable (the diagonal values) exceeded the square root of other items (70). In addition, the recorded HTMT values were below 0.9, which empirically established effective distinctions.

**Table 3:** Discriminant validity.

	**HCS**	**HMO**	**PCT**	**PCM**	**PUS**	**PTA**	**PPP**	**PPV**	**ITU**	**ADT**
**Fornell-Larcker criterion**										
HCS	0.849									
HMO	0.593	0.863								
PCT	0.537	0.676	0.928							
PCM	0.377	0.505	0.770	0.910						
PUS	0.329	0.365	0.556	0.513	0.915					
PTA	0.415	0.481	0.621	0.613	0.482	0.920				
PPP	0.481	0.525	0.590	0.537	0.465	0.635	0.887			
PPV	0.429	0.429	0.616	0.609	0.474	0.627	0.654	0.926		
ITU	0.422	0.492	0.723	0.725	0.569	0.723	0.659	0.759	0.938	
ADT	0.272	0.354	0.572	0.633	0.395	0.677	0.503	0.707	0.771	1.000
**Heterotrait-Monotrait ratio**										
HCS										
HMO	0.648									
PCT	0.569	0.719								
PCM	0.394	0.534	0.805							
PUS	0.350	0.391	0.581	0.536						
PTA	0.441	0.511	0.648	0.643	0.504					
PPP	0.520	0.569	0.623	0.566	0.492	0.672				
PPV	0.457	0.456	0.643	0.636	0.495	0.656	0.693			
ITU	0.445	0.520	0.750	0.755	0.591	0.753	0.693	0.788		
ADT	0.280	0.365	0.584	0.648	0.403	0.693	0.520	0.722	0.785	

*HCS, Health consciousness; HMO, Health motivation; PCT, Perceived compatibility; PCM, Perceived critical mass; PUS, Perceived usefulness; PTA, Perceived technology accuracy; PPP, Perceived privacy protection; PPV, Perceived product value; ITU, Intention to use eDoctor Apps; ADT, Adoption of eDoctor Apps*.

*Source: Author's data analysis*.

As noted in Table 4, all loading values were more than 0.5 and exceeded the cross-loading values. These results further established the discriminant validity of all items used in this study (53).

**Table 4:** Loading and cross loadings.

**Items**	**HCS**	**HMO**	**PCT**	**PCM**	**PUS**	**PTA**	**PPP**	**PPV**	**ITU**	**ADT**
HCS1	0.818	0.398	0.420	0.310	0.270	0.346	0.404	0.362	0.335	0.232
HCS2	0.871	0.510	0.444	0.345	0.256	0.347	0.400	0.370	0.355	0.249
HCS3	0.875	0.481	0.453	0.252	0.281	0.330	0.400	0.358	0.347	0.195
HCS4	0.848	0.453	0.403	0.218	0.254	0.305	0.369	0.310	0.299	0.166
HCS5	0.831	0.632	0.533	0.433	0.319	0.413	0.450	0.404	0.428	0.288
HMO1	0.585	0.811	0.512	0.321	0.293	0.347	0.420	0.309	0.340	0.208
HMO2	0.522	0.835	0.572	0.413	0.315	0.396	0.440	0.351	0.401	0.288
HMO3	0.496	0.891	0.609	0.502	0.322	0.455	0.470	0.396	0.462	0.354
HMO4	0.513	0.887	0.610	0.453	0.316	0.425	0.459	0.381	0.458	0.323
HMO5	0.471	0.889	0.606	0.467	0.328	0.439	0.473	0.402	0.445	0.331
PCT1	0.483	0.606	0.917	0.688	0.511	0.549	0.519	0.552	0.641	0.507
PCT2	0.519	0.633	0.936	0.709	0.515	0.579	0.543	0.568	0.673	0.535
PCT3	0.507	0.656	0.941	0.726	0.508	0.583	0.559	0.591	0.683	0.539
PCT4	0.500	0.636	0.922	0.713	0.536	0.577	0.564	0.570	0.676	0.532
PCT5	0.484	0.607	0.924	0.738	0.509	0.591	0.548	0.577	0.680	0.540
PCM1	0.345	0.464	0.721	0.922	0.444	0.570	0.476	0.551	0.646	0.573
PCM2	0.329	0.454	0.699	0.928	0.435	0.553	0.483	0.546	0.654	0.577
PCM3	0.328	0.441	0.680	0.925	0.438	0.542	0.451	0.517	0.633	0.574
PCM4	0.321	0.427	0.660	0.917	0.470	0.528	0.473	0.530	0.630	0.552
PCM5	0.385	0.503	0.734	0.857	0.535	0.590	0.548	0.615	0.722	0.595
PUS1	0.292	0.339	0.491	0.483	0.909	0.394	0.389	0.411	0.482	0.340
PUS2	0.302	0.341	0.548	0.534	0.917	0.466	0.463	0.461	0.549	0.393
PUS3	0.326	0.353	0.521	0.468	0.916	0.469	0.463	0.465	0.551	0.390
PUS4	0.300	0.327	0.495	0.425	0.914	0.420	0.396	0.401	0.491	0.321
PUS5	0.282	0.307	0.483	0.431	0.917	0.446	0.408	0.421	0.522	0.355
PTA1	0.380	0.441	0.569	0.549	0.453	0.909	0.577	0.551	0.634	0.605
PTA2	0.385	0.450	0.589	0.569	0.466	0.922	0.591	0.594	0.676	0.619
PTA3	0.395	0.456	0.585	0.571	0.457	0.934	0.615	0.606	0.687	0.635
PTA4	0.385	0.449	0.561	0.564	0.421	0.924	0.574	0.567	0.664	0.626
PTA5	0.366	0.414	0.550	0.567	0.417	0.908	0.560	0.564	0.663	0.626
PPP1	0.425	0.478	0.552	0.513	0.428	0.597	0.915	0.594	0.620	0.483
PPP2	0.425	0.472	0.540	0.528	0.421	0.598	0.910	0.609	0.624	0.490
PPP3	0.441	0.461	0.522	0.471	0.412	0.553	0.910	0.576	0.587	0.424
PPP4	0.419	0.452	0.504	0.392	0.409	0.516	0.826	0.547	0.532	0.390
PPP5	0.424	0.467	0.494	0.466	0.392	0.545	0.871	0.572	0.552	0.437
PPV1	0.396	0.404	0.568	0.596	0.422	0.584	0.606	0.914	0.675	0.638
PPV2	0.438	0.419	0.585	0.540	0.442	0.592	0.632	0.914	0.692	0.627
PPV3	0.415	0.438	0.596	0.592	0.450	0.603	0.622	0.940	0.718	0.673
PPV4	0.362	0.352	0.557	0.554	0.438	0.559	0.582	0.929	0.710	0.663
PPV5	0.380	0.374	0.548	0.540	0.441	0.567	0.590	0.931	0.716	0.671
ITU1	0.398	0.469	0.681	0.662	0.530	0.684	0.626	0.714	0.933	0.708
ITU2	0.378	0.459	0.678	0.685	0.525	0.668	0.612	0.700	0.928	0.723
ITU3	0.396	0.466	0.685	0.687	0.542	0.695	0.634	0.720	0.956	0.729
ITU4	0.399	0.442	0.668	0.686	0.536	0.669	0.598	0.705	0.938	0.741
ITU5	0.410	0.474	0.678	0.682	0.535	0.677	0.624	0.720	0.935	0.717
ADT	0.272	0.354	0.572	0.633	0.395	0.677	0.503	0.707	0.771	1.000

*HCS, Health consciousness; HMO, Health motivation; PCT, Perceived compatibility; PCM, Perceived critical mass; PUS, Perceived usefulness; PTA, Perceived technology accuracy; PPP, Perceived privacy protection; PPV, Perceived product value; ITU, Intention to use eDoctor Apps; ADT, Adoption of eDoctor Apps*.

*Source: Author's data analysis*.

### Hypothesis Testing

This section first discusses the potential of multicollinearity. As shown in Table 2, VIF values ranged between 1.563 and 3.830, which did not exceed the threshold value of 5, as recommended by Hair et al. (69). Thus, the multicollinearity issue was disregarded, and the correlation structure in the measurement model was considered.

This study evaluated the structural model using predictive relevance (*Q*^2^), coefficient of determination (*R*^2^), and effect size (*f*^2^). The blindfolding method was used to examine the correlation structure. Referring to Table 5, the values of *Q*^2^ of ITU (0.62) and ADT (0.62) were greater than zero, indicating the presence of predictive. The values of *R*^2^ ranges from 0 to 1; a higher value indicates better explanatory power of the model. Based on the recommendations by Hair et al. (53), and considering the background of this study, the recorded *R*^2^ values of ITU (*R*^2^ = 0.71) and ADT (*R*^2^ = 0.63) were higher than 0.50, indicating high explanatory power of the model for TIU and ADT. Considering the recommendations by Hair et al. (69) and the context of moderation, with the omission of the interpretation of *R*^2^ from the model, *f*^2^ is recommended to explain the contribution of moderation to the endogenous structure. In this study, referring to the threshold value proposed by Kenny (71), PPV (*f*^2^ = 0.10) contributed medium moderating effect on the relationship between ITU and ADT.

**Table 5:** Hypothesis testing.

**Hypothesis**		**Beta**	**CI (Min)**	**CI (Max)**	**t**	***p***	***R^**2**^***	***f^**2**^***	***Q^**2**^***	**Decision**
**Factors affecting the intention to use eDoctor Apps**										
H_1_	HCS → ITU	0.00	−0.05	0.04	0.12	0.45		0.00		Reject
H_2_	HMO → ITU	−0.06	−0.12	−0.01	1.78	0.04		0.01		Reject
H_3_	PCT → ITU	0.21	0.13	0.30	4.16	0.00		0.04		Supported
H_4_	PCM → ITU	0.25	0.19	0.32	6.26	0.00	0.71	0.08	0.62	Supported
H_5_	PUS → ITU	0.11	0.07	0.17	3.63	0.00		0.03		Supported
H_6_	PTA → ITU	0.29	0.22	0.36	6.33	0.00		0.13		Supported
H_7_	PPP → ITU	0.20	0.12	0.27	4.63	0.00		0.06		Supported
**Factors affecting the adoption of eDoctor Apps**										
H_8_	ITU → ADT	0.54	0.44	0.64	8.90	0.00	0.63	0.35	0.62	Supported
**Mediating effect of intention to use eDoctor Apps (H**_**9M**_ **)**										
PCT → ITU → ADT		0.12	0.07	0.17	3.71	0.00				Mediates
PCM → ITU → ADT		0.14	0.10	0.19	4.84	0.00				Mediates
PUS → ITU → ADT		0.06	0.04	0.10	3.37	0.00				Mediates
PTA → ITU → ADT		0.16	0.11	0.21	4.82	0.00				Mediates
PPP → ITU → ADT		0.11	0.07	0.14	4.52	0.00				Mediates
**Moderating effect of perceived product value**										
	PPV → ADT	0.30	0.20	0.39	5.02	0.00		0.10		No moderation
H_10_	PPV*ITU → ADT	−0.02	−0.05	0.01	0.96	0.17				

*HCS, Health consciousness; HMO, Health motivation; PCT, Perceived compatibility; PCM, Perceived critical mass; PUS, Perceived usefulness; PTA, Perceived technology accuracy; PPP, Perceived privacy protection; PPV, Perceived product value; ITU, Intention to use eDoctor Apps; ADT, Adoption of eDoctor Apps*.

*Source: Author's data analysis*.

Table 5 presents the results of the testing of hypotheses in this study. PCT (H_3_: β = 0.21, *p* < 0.05), PCM (H_4_: β = 0.25, *p* < 0.05), PUS (H_5_: β = 0.11, *p* < 0.05), PTA (H_6_: β = 0.29, *p* < 0.05), and PPP (H_7_: β = 0.20, *p* < 0.05) were found to significantly and positively influence ITU. Meanwhile, ITU (H_8_: β = 0.54, *p* < 0.05) had significant, positive effect on ADT. This study also tested the significance level with 90% confidence interval. The confidence intervals of H_3_, H_4_, H_5_, H_6_, H_7_, and H_8_ in Table 5 did not contain zero between the 5% CI and 95% CI, indicating that these hypotheses were accepted.

The relationship between HCS and ITU (H_1_) was found positive (H_1_: β = 0.00, *p* = 0.45), suggesting the positive influence of HCS on ITU. However, the effect was not statistically significant. On the other hand, HMO (β = −0.06, *p* < 0.05) had statistically significant impact on ITU, but the recorded path coefficient was negative. Thus, H_2_ was rejected.

In terms of mediating effects, the results in Table 5 revealed that ITU contributed significant and positive mediation effects on the relationships of PCT (β = 0.12, *p* < 0.05), PCM (β = 0.14, *p* < 0.05), PUS (β = 0.06, *p* < 0.05), PTA (β = 0.16, *p* < 0.05), PPP (β = 0.11, *p* < 0.05) with ADT. For these mediating effects, the 90% confidence interval did not contain zero between 5% CI and 95% CI, with statistically significant weight. Based on these obtained results, H_9M_ was supported.

Finally, this study tested the moderating effect of PPV on the relationship between ITU and ADT. The obtained results confirmed that the moderating effect of PPV on this particular relationship was not statistically significant at a significance level of 5%.

### Multi-Group Analysis

The study assessed the measurement invariance using the measurement invariance of composite models (MICOM) procedure for two groups: (1) Group 1: Completed secondary/diploma/technical diploma; (2) Group 2: Bachelor's degree, Master's degree, and doctorate degree. The permutation *p-*values for all constructs in this study exceeded 0.05, which confirmed partial measurement invariance. Therefore, the study was able to compare the path coefficients between both groups using PLS-MGA. The results in Table 6 revealed no significant differences between both groups based on education level in all hypothesized relationships.

**Table 6:** Multi-group analysis.

**Associations**		**Secondary/diploma/technical diploma (*****N*** **=** **190)**		**Bachalor/master/doctorate degree (*****N*** **=** **771)**		**Difference**		**Decision**
		**Beta**	***p*-value**	**Beta**	***p*-value**	**Beta**	***p*-value**	
H_1_	HCS → ITU	−0.055	0.213	0.019	0.257	−0.074	0.151	No difference
H_2_	HMO → ITU	−0.088	0.166	−0.052	0.062	−0.035	0.354	No difference
H_3_	PCT → ITU	0.199	0.117	0.218	0.000	−0.019	0.460	No difference
H_4_	PCM → ITU	0.234	0.032	0.246	0.000	−0.012	0.468	No difference
H_5_	PUS → ITU	0.149	0.053	0.101	0.000	0.047	0.343	No difference
H_6_	PTA → ITU	0.291	0.025	0.287	0.000	0.004	0.464	No difference
H_7_	PPP → ITU	0.198	0.110	0.200	0.000	−0.003	0.481	No difference
H_8_	ITU → ADT	0.666	0.000	0.520	0.000	0.147	0.119	No difference
**Associations**		**Male** **(*****N*** **=** **425)**		**Female** **(*****N*** **=** **536)**		**Difference**		**Decision**
		**Beta**	***p*** **-value**	**Beta**	***p*** **-value**	**Beta**	***p*** **-value**	
H_1_	HCS → ITU	0.013	0.318	−0.005	0.458	0.018	0.374	No difference
H_2_	HMO → ITU	−0.028	0.270	−0.083	0.048	0.055	0.210	No difference
H_3_	PCT → ITU	0.269	0.001	0.174	0.003	0.095	0.181	No difference
H_4_	PCM → ITU	0.235	0.000	0.250	0.000	−0.015	0.424	No difference
H_5_	PUS → ITU	0.077	0.002	0.134	0.003	−0.057	0.151	No difference
H_6_	PTA → ITU	0.231	0.000	0.336	0.000	−0.105	0.110	No difference
H_7_	PPP → ITU	0.251	0.000	0.149	0.006	0.101	0.102	No difference
H_8_	ITU → ADT	0.597	0.000	0.514	0.000	0.083	0.249	No difference
**Associations**		**Using healthcare monitoring device** **(*****N*** **=** **280)**		**Never used healthcare monitoring device** **(*****N*** **=** **680)**		**Difference**		**Decision**
		**Beta**	***p*** **-value**	**Beta**	***p*** **-value**	**Beta**	***p*** **-value**	
H_1_	HCS → ITU	0.047	0.204	−0.017	0.307	0.064	0.157	No difference
H_2_	HMO → ITU	0.067	0.225	−0.075	0.025	0.141	0.073	No difference
H_3_	PCT → ITU	0.139	0.065	0.220	0.000	−0.081	0.227	No difference
H_4_	PCM → ITU	0.216	0.001	0.260	0.000	−0.043	0.306	No difference
H_5_	PUS → ITU	0.069	0.034	0.123	0.002	−0.055	0.168	No difference
H_6_	PTA → ITU	0.236	0.000	0.296	0.000	−0.059	0.240	No difference
H_7_	PPP → ITU	0.243	0.000	0.182	0.000	0.060	0.233	No difference
H_8_	ITU → ADT	0.533	0.000	0.562	0.000	−0.030	0.437	No difference

Following that, this study assessed the measurement invariance between another two groups using the MICOM procedure: (1) Group 1: Male; (2) Group 2: Female. The permutation *p*-values for all constructs exceeded 0.05, which confirmed partial measurement invariance. Therefore, the study was able to compare the path coefficients between these two groups using PLS-MGA. The results in Table 6 revealed no significant differences between both groups based on gender in all hypothesized relationships.

Finally, this study assessed the measurement invariance between the following two groups using the MICOM procedure: (1) Group 1: Have used healthcare monitoring device; (2) Group 2: Never use healthcare monitoring device. The permutation *p*-values for all constructs exceeded 0.05, which confirmed partial measurement invariance. Therefore, the study was able to compare the path coefficients between these two groups using PLS-MGA. The results in Table 6 revealed no significant differences between these two groups based on the use of healthcare monitoring device in all hypothesized relationships.

#### Artificial Neural Network Analysis

For the current study, the ANN analysis was performed to estimate the impact of the input variables on the outcomes. Two ANN models were used in this study to assess ITU and ADT. Addressing the over-estimation issue, a ten-fold analysis was performed. Model A (see Table 7) showed that the obtained RMSE scores for training and testing of ANN analysis were close and showed higher predictive accuracy. For Model B, the predictive accuracy was achieved, as the obtained RMSE scores for training and testing of ANN analysis were close. The goodness-of-fit outcomes of Model B described that the input variables can predict ADT, with 99.2% (72).

**Table 7:** RMSE values of artificial neural networks (*N* = 961).

**Model A: factors affecting the ITU**						**Model B: factors affecting the adoption of eDoctor Apps**				
**Network**	**Sample size (training)**	**Sample size (testing)**	**RMSE (Training)**	**RMSE** **(testing)**	**RMSE (difference)**	**Sample size (training)**	**Sample size (testing)**	**RMSE** **(training)**	**RMSE (testing)**	**RMSE** **(difference)**
1	654	307	0.316	0.401	0.085	671	290	0.402	0.482	0.080
2	679	282	0.387	0.348	0.038	678	283	0.424	0.375	0.049
3	661	300	0.320	0.411	0.091	665	296	0.409	0.441	0.032
4	674	287	0.345	0.339	0.006	655	306	0.436	0.400	0.036
5	656	305	0.370	0.335	0.035	690	271	0.428	0.399	0.029
6	673	288	0.322	0.375	0.052	672	289	0.406	0.414	0.007
7	674	287	0.326	0.324	0.002	685	276	0.418	0.428	0.011
8	677	284	0.330	0.360	0.030	685	276	0.426	0.398	0.028
9	683	278	0.335	0.382	0.046	679	282	0.427	0.368	0.059
10	650	311	0.332	0.329	0.003	661	300	0.393	0.493	0.100
		Mean	0.338	0.360	0.039		Mean	0.417	0.420	0.043
	Standard deviation		0.023	0.032	0.032	Standard deviation		0.014	0.042	0.029

*Source: Author's data analysis*.

The multi-factor sensitivity analysis was conducted for this study to determine the critical factors of ITU (Model A) and ADT (Model B). Two ANN models were employed to evaluate the critical factors in this study. As presented in Table 8, the results of sensitivity analysis for Model A depicted PTA, PUS, PCM, PCT, PPP, HMO, and HCS as the most crucial factors that affect ITU. The results of sensitivity analysis for Model B suggested ITU as the most prominent factors that affect ADT.

**Table 8:** Sensitivity analysis.

	**Model A: factors** **affecting ITU**							**Model B: factors affecting ADT**	
**Network**	**HCS**	**HMO**	**PCT**	**PCM**	**PUS**	**PTA**	**PPP**	**ITU**	PPV*ITU
1	0.082	0.165	0.138	0.113	0.157	0.237	0.107	0.780	0.220
2	0.025	0.055	0.124	0.247	0.135	0.321	0.093	0.515	0.485
3	0.080	0.101	0.138	0.100	0.196	0.282	0.103	0.680	0.320
4	0.039	0.030	0.139	0.155	0.183	0.378	0.077	0.718	0.282
5	0.055	0.105	0.100	0.139	0.183	0.281	0.137	0.410	0.590
6	0.018	0.108	0.130	0.096	0.149	0.407	0.092	0.690	0.310
7	0.019	0.096	0.112	0.160	0.144	0.385	0.084	0.758	0.242
8	0.054	0.080	0.109	0.149	0.183	0.362	0.063	0.721	0.279
9	0.050	0.105	0.160	0.143	0.115	0.269	0.157	0.737	0.263
10	0.058	0.103	0.095	0.131	0.183	0.348	0.082	0.677	0.323
Mean importance	0.048	0.095	0.125	0.143	0.163	0.327	0.100	0.669	0.331

*HCS, Health consciousness; HMO, Health motivation; PCT, Perceived compatibility; PCM, Perceived critical mass; PUS, Perceived usefulness; PTA, Perceived technology accuracy; PPP, Perceived privacy protection; PPV, Perceived product value; ITU, Intention to use eDoctor Apps; ADT, Adoption of eDoctor Apps*.

*Source: Author's data analysis*.

## Discussion

This study found no positive relationship between health consciousness and intention to use. Therefore, H_1_ was not supported. However, Srivastava et al. (10) supported the positive relationship between health consciousness and intention to use. In another study that involved online food ordering behavior, Kaur et al. (9) showed no positive relationship between health consciousness and intention to use and further noted that consumers with higher health consciousness may be more inclined to read online reviews of takeaway restaurants. In this case, when a restaurant has positive reviews, consumers may abandon the need to purchase ingredients to cook and choose to order takeaway from the restaurant instead. Surprisingly, the current study found a significant, negative relationship between health consciousness and intention to use. A logical explanation for this particular finding involves the lack of examination items currently provided by eDoctor apps, such as CT, MRI, blood test, and COVID-19 test. Individuals with high health consciousness who experience medical problems may be inclined to rely on the results of physical examination, which involves the need to visit an offline hospital for a physical examination to ensure the accuracy and effectiveness of medical diagnosis.

Besides that, the results of this study showed no effect of health motivation on intention to use. Thus, H_2_ was rejected. Previous studies often discussed the relationship between health motivation and behavioral intention; certain studies suggested a positive relationship between health motivation and behavioral intention (8, 73). Several other studies reported different findings. For instance, Nguyen et al. (74) identified health motivation as an insignificant predictor of behavioral intention. The results of the current study can be explained based on the following notion: health-driven individuals pay more attention to health-related information, such as more detailed functional requirements and process requirements (75). With the increase in health motivations, consumers have become skeptical of product functional claims (11). Furthermore, the majority of the respondents (80.1%) in this study attained higher education level, at least a Bachelor's degree. Individuals with higher education level typically have more independent thinking and are more likely to think before they purchase any products. They may carry out a detailed examination of the product features, rather than just relying on product claims.

On the other hand, the hypothesis (H_3_) on the significant and positive relationship between perceived compatibility and intention to use in this study was supported, which confirmed the findings of prior studies (20, 57, 76, 77). The current research offered empirical evidence for future eDoctor app research. Users are getting used to interacting with items that reflect their values, beliefs, and way of life. To put it another way, if eDoctor Apps can be created in a way that people are acquainted with, this technology will be more readily adopted.

The obtained results also reported the significant, positive impact of perceived critical mass on intention to use (H_4_), which supported the findings of prior studies on communication technology (24) and Internet services (78). In previous studies, perceived critical mass was typically discussed as a pre-factor of trust (79) or satisfaction (80). The current study clearly enriched the practical and research significance on the influence of perceived critical mass. As healthcare technology becomes more widely known and adopted, the public's attitude toward eDoctor Apps will be influenced by others, leading to more active use of the product.

The results of this study supported the significant and positive relationship between perceived usefulness and intention to use (H_5_), which confirmed the findings of prior studies. Ambalov (31) conducted a detailed analysis on perceived usefulness from the direction of information technology, confirming the importance of perceived usefulness as an improvement in information technology. Sreelakshmi and Prathap (28) confirmed the significant impact of perceived usefulness on the intention to use mobile-based payments during COVID-19. On a similar note, Liu et al. (81) pointed out that perceived usefulness enhances the intention of using mobile health services. In another study on wearable medical device usage intention among Koreans, perceived usefulness was confirmed to have significant impact on the intention to use wearable medical devices (82). Likewise, in another study related to mobile health services, Zhao et al. (83) proposed the significant correlation between perceived usefulness and the intention to use mobile health services. Leung and Chen (29) reported similar findings on e-health use intention in Hong Kong. Previous studies of different industries and populations (of different countries) supported the current study's results of this study. This study once again provided evidence that support the significant, positive relationship between perceived usefulness and intention to use. As can be seen, one of the reasons the public uses eDoctor Apps is to accomplish the goal of health maintenance more efficiently and conveniently. Particularly in light of the scarcity of medical resources during COVID-19, perceived usefulness is a critical factor in the public's intention to use eDoctor Apps.

In addition, this study demonstrated the significant, positive relationship between perceived technology accuracy and intention to use (H_6_), which was found consistent with the results of previous studies on online banking (84) and mHealth services. The perceived accuracy of technology will draw a bigger user base to eDoctor Apps as healthcare technology continues to progress. Similarly, this study obtained adequate evidence to support the significant and positive relationship between perceived privacy protection and intention to use (H_7_), which was found consistent with the results of previous studies (10). In terms of theoretical support, intention to use is regarded the core factor of TAM (85, 86) and UTAUT (87–89). With that, the intention to use a technology can predict users' actual use of the technology. In this study, intention to use was found to contribute significant and positive impact on adoption of eDoctor Apps (H_8_), which was found consistent with the results of previous studies. Due to the advancement of information technology, numerous data leakage incidents occur on a regular basis, and the harm caused by the leakage of personal health information may result in unpredictable losses for individuals. As a result, privacy and security may become a primary consideration for users prior to adopting eDoctor Apps.

Considering the consistency of the previous hypothesis on intention to use as the motivating factor of adoption, intention to use in this study was found to contribute significant, positive mediating effects on the relationships of perceived compatibility, perceived critical mass, perceived usefulness, perceived technology accuracy, and perceived privacy protection with adoption of eDoctor Apps. The obtained results of this study confirmed the findings of previous studies; if users have more cognition about perceived compatibility (90, 91), perceived critical mass (92), perceived usefulness (93, 94), perceived technology accuracy (94), and perceived privacy protection (95), they are more likely to have positive intention and consider adoption.

Lastly, this study examined the moderating effect of perceived product value on the relationship between intention to use and adoption of eDoctor Apps. The obtained results confirmed the significant, positive moderating effect of perceived product value on this particular relationship. It was deemed not surprising, as product value is evidently one of the important characteristics of a product (96). In many cases, perceived value yields positive effects, such as higher customer satisfaction (97), behavioral intention (96, 98), and actual behavior (adoption) (96). With that, this study confirmed the translation of intention to use into adoption of eDoctor Apps via elevated perceived product value for the eDoctor apps.

## Implications

### Theoretical Implications

Technology adoption is substantially based on the technical attributes and the personal inclination toward technology. The current study demonstrated that health-related technology adoption significantly emerges from the technological attributes of health-related technologies, which added to the related theoretical domain (99). The current study presented evidence that individual health consciousness and motivation are not prominent in the development of intention to use health-related technologies. Moreover, the current study attempted to encompass UTAUT with healthcare predispositions, such as health consciousness and motivation. Personal inclination is an essential predictor in the formation of one's intention to use technology (8). However, the current study's results suggested low health inclinations among Chinese adults, which restrict the formation of their intention to use health-related technologies. Additionally, the current study extended the explaining power of UTAUT that technology usefulness and accuracy are the most significant factors that influence the formation of the intention to use health-related technologies for personal healthcare. The current work also offered pertinent evidence on the significance of perceived technology value in the adoption of technology and its moderating effect on the relationship between the intention to use health-related technologies and the adoption of health-related technologies.

### Practical Implications

The current study offered three practical implications. Firstly, the healthcare technology industry and management must improve the attributes of privacy and compatibility in order to empower the adoption of health-related technologies. The current study's results demonstrated low health-related technology adoption among the masses. Technology firms should consider offering free samples and free usage of healthcare technologies to harness the public attitude and form the intention to use these technologies (100). Simultaneously, actively collaborate with medical institutions, host health lectures, and maintain extensive contacts with relevant medical academic groups and academic journals to raise user awareness of innovative medical and health-care products, thereby improving the public's perception of medical technology's value (100). Secondly, healthcare technologies present a set of attributes that can form the product value, specifically on whether the product is viable or worthwhile to be used to manage personal health. Firms that develop and sell these technologies need to inform and promote the values of personal healthcare products and the adoption of personal healthcare technologies. The company may also wish to establish an independent technical guidance department to provide detailed explanations and guidance to customers regarding product functions, attributes, values, user guides, and other related information, in order to ensure that customers have a thorough understanding of the product (101, 102). Lastly, the massive promotion of health consciousness and motivation is also necessary to persuade more consumers to start taking personal health at a personal level. The lack of personal health consciousness and motivation encourages the dearth of personal healthcare and may eventually burden the public healthcare system (103). Policymakers should consider formulating public awareness and personal healthcare responsiveness campaigns that can empower the general public to invest in and mindfully take care of their personal health. These campaigns can target a variety of demographics, including the elderly, who have a high prevalence of chronic diseases, and young people, who are concerned about daily health care and are receptive to new technologies.

## Conclusions

Overall, the current study examined the formation of intention and adoption of eDoctor apps among Chinese adults in relation to personal health inclinations and technological attributes. The study's results confirmed the lack of personal health inclinations among Chinese adults, restricting the formation of the intention to use the healthcare technology. The technology-level features of compatibility, usefulness, accuracy, and privacy protection meaningfully instigate their intention to use the technology. Healthcare technologies are not massively penetrated among Chinese consumers, limiting their intention to use eHealth services. The mass adoption of eHealth services based on the development of precise product value can harness the adoption and curtail the burden on the healthcare system. The future of healthcare system requires the participation of the general public to shift toward upkeeping personal health and use eHealth services that facilitate the public health system. The mass adoption of eHealth services promotes the formation of intention and subsequently, the adoption of healthcare technologies. Technology value can nurture healthcare technologies and collaboratively promote a viable healthcare system.

## Data Availability Statement

The original contributions presented in the study are included in the article/Supplementary Material, further inquiries can be directed to the corresponding author/s.

## Ethics Statement

Ethical review and approval was not required for the study on human participants in accordance with the local legislation and institutional requirements. The patients/participants provided their written informed consent to participate in this study.

## Author Contributions

NH, MM, AS, and ZM: conceptualization, methodology, instrument, and writing—original draft. QY and AA: data collection, formal analysis, and writing—editing. All authors contributed to the article and approved the submitted version.

## Conflict of Interest

The authors declare that the research was conducted in the absence of any commercial or financial relationships that could be construed as a potential conflict of interest.

## Publisher's Note

All claims expressed in this article are solely those of the authors and do not necessarily represent those of their affiliated organizations, or those of the publisher, the editors and the reviewers. Any product that may be evaluated in this article, or claim that may be made by its manufacturer, is not guaranteed or endorsed by the publisher.

## Supplementary Material

Supplementary Table 1 Data file - Predicting the mass adoption of eDoctor apps.

## Appendix

**Table A1:** Survey instrument.

*Health Consciousness (HCS)*	
HCS1.	I think my health depends on how well I take care of myself.
HCS2.	I am actively engaged in the prevention of disease and illness.
HCS3.	I think taking preventive measures help to stay healthy.
HCS4.	Living a healthy life is important to me.
HCS5.	I am constantly examining my health.
*Health Motivation (HMO)*	
HMO1.	I usually value my health.
HMO2.	I have good knowledge to prevent health issues.
HMO3.	I try to prevent health problems before I feel any symptoms.
HMO4.	I try to protect myself against health hazards I hear about.
HMO5.	I am concerned about health hazards and try to take action to prevent them
*Perceived Compatibility (PCT)*	
PCT1.	Using the eDoctor App would be compatible with my lifestyle.
PCT2.	I think that using the eDoctor App would fit well with the way I work and live.
PCT3.	Using the eDoctor App is compatible with all aspects of my current health care management at my personal level.
PCT4.	I think using the eDoctor App suits my way of managing health at home.
PCT5.	I think the eDoctor App is very much compatible with my lifestyle.
*Perceived Critical Mass (PCM)*	
PCM1.	Most people in my neighborhood are using the eDoctor App.
PCM2.	Many people to whom I usually communicate are using the eDoctor App.
PCM3.	Most people in my community are using the eDoctor App frequently.
PCM4.	I know many people having health issues are using the eDoctor App regularly.
PCM5.	eDoctor App devices are gaining popularity.
*Perceived Usefulness (PUS)*	
PUS1.	Using the eDoctor App enables me to check my health condition quickly.
PUS2.	Using the eDoctor App makes it easier to check my health condition.
PSU3.	Using the eDoctor App save my time and effort.
PUS4.	eDoctor App is beneficial to manage health.
PUS5.	eDoctor App is useful to check my health condition.
*Perceived Technology Accuracy (PTA)*	
PTA1.	I think I can rely on the health services provided by eDoctor Apps.
PTA2.	I think the eDoctor App delivers consistent results over time.
PTA3.	I think eDoctor App have good working standards continuously.
PTA4.	I think eDoctor Apps are reliable.
PTA5.	I feel confident that eDoctor Apps are offering error-free results.
*Perceived Privacy Protection (PPP)*	
PPP1.	It would be risky to disclose my personal health information to vendors providing eDoctor Apps.
PPP2.	There would be a high potential for loss associated with disclosing my personal health information to vendors providing eDoctor Apps.
PPP3.	There would be too much uncertainty associated with giving my personal health information to vendors providing eDoctor Apps.
PPP4.	Disclosing personal information to a third party is risky.
PPP5.	I feel a loss of control over my personal information by using eDoctor Apps.
*Perceived Product Value (PPV)*	
PPV1.	eDoctor Apps offer good value for money.
PPV2.	Using eDoctor Apps are beneficial
PPV3.	Using eDoctor Apps are valuable to me.
PPV4.	I think the eDoctor App is worthwhile.
PPV5.	Overall, using the eDoctor App delivers good value to me.
*Intention to use EDoctor Apps (ITU)*	
ITU1.	I intend to use eDoctor apps to manage my health in the future.
ITU2.	I will always try to use eDoctor apps to manage my health in my daily life in the future.
ITU3.	I plan to use eDoctor apps frequently to manage my health in the future.
ITU4.	I would be willing to develop a habit to use eDoctor apps soon.
ITU5.	I predict I will use eDoctor apps to manage my health information.
*Adoption of EDoctor Apps (ADT)*	
ADT1.	How often do you use eDoctor Apps?

## References

[1] Akhtar SM Nazir M Saleem K Ahmad RZ Javed ARS Band S. A multi-agent formalism based on contextual defeasible logic for healthcare systems. Front Public Health. (2022) 10:849185. 10.3389/fpubh.2022.849185 35309219 PMC8927623

[2] Esmaeilzadeh P. How does IT identity affect individuals' use behaviors associated with personal health devices (PHDs)? An empirical study. Inform Manage. (2021) 58:103313. 10.1016/j.im.2020.103313

[3] He D Gu Y Shi Y Wang M Lou Z Jin C. COVID-19 in China: the role and activities of internet-based healthcare platforms. Global Health Med. (2020) 2:89–95. 10.35772/ghm.2020.01017 33330783 PMC7731100

[4] Saleem K Saleem M Zeeshan R Javed AR Alazab M Gadekallu TR. Situation-aware BDI reasoning to detect early symptoms of Covid 19 using Smartwatch. IEEE Sens J. (2022). 10.1109/JSEN.2022.3156819 PMC9983688 36913222

[5] Rowland SP Fitzgerald JE Holme T Powell J McGregor A. What is the clinical value of mHealth for patients? NPJ Dig Med. (2020) 3:4. 10.1038/s41746-019-0206-x 31970289 PMC6957674

[6] Molina MD Myrick JG. The ‘how' and ‘why' of fitness app use: investigating user motivations to gain insights into the nexus of technology and fitness. Sport Soc. (2020) 24:1233–48. 10.1080/17430437.2020.1744570

[7] Gao Y Li H Luo Y. An empirical study of wearable technology acceptance in healthcare. Indus Manage Data Syst. (2015) 115:1704–23. 10.1108/IMDS-03-2015-0087

[8] Kumphong J Satiennam T Satiennam W. The determinants of motorcyclists helmet use: urban arterial road in Khon Kaen city, Thailand. J Safety Res. (2018) 67:93–7. 10.1016/j.jsr.2018.09.011 30553434

[9] Kaur P Dhir A Talwar S Ghuman K. The value proposition of food delivery apps from the perspective of theory of consumption value. Int J Contemp Hosp Manage. (2021) 33:1129–59. 10.1108/IJCHM-05-2020-0477

[10] Srivastava NK Chatterjee N Subramani A Akbar Jan N Singh PK. Is health consciousness and perceived privacy protection critical to use wearable health devices? Extending the model of goal-directed behavior. Benchmark Int J. (2021). 10.1108/BIJ-12-2020-0631

[11] Verbeke W Scholderer J Lähteenmäki L. Consumer appeal of nutrition and health claims in three existing product concepts. Appetite. (2009) 52:684–92. 10.1016/j.appet.2009.03.007 19501767

[12] Kim T Chiu W. Consumer acceptance of sports wearable technology: the role of technology readiness. Int J Sports Mark Sponsor. (2019) 20:109–26. 10.1108/IJSMS-06-2017-0050

[13] Venkatesh V Thong JY Chan FK Hu PJ Brown SA. Extending the two-stage information systems continuance model: Incorporating UTAUT predictors and the role of context. Inform Syst J. (2011) 21:527–55. 10.1111/j.1365-2575.2011.00373.x

[14] Dellande S Gilly MC Graham JL. Gaining compliance and losing weight: the role of the service provider in health care services. J Mark. (2004) 68:78–91. 10.1509/jmkg.68.3.78.34764

[15] Sakib MN Zolfagharian M Yazdanparast A. Does parasocial interaction with weight loss vloggers affect compliance? The role of vlogger characteristics, consumer readiness, and health consciousness. J Retail Consum Serv. (2020) 52:101733. 10.1016/j.jretconser.2019.01.002

[16] Moorman C Matulich E. A model of consumers' preventive health behaviors: the role of health motivation and health ability. J Consu Res. (1993) 20:208. 10.1086/209344

[17] Becker HM. The health belief model and sick role behavior. Health Educ Monographs. (1974) 2:409–19. 2214655

[18] Sohail MS Al-Jabri IM. Attitudes towards mobile banking: are there any differences between users and non-users? Behav Inf Technol. (2014) 33:335–44. 10.1080/0144929X.2013.763861

[19] Amaro S Duarte P. An integrative model of consumers' intentions to purchase travel online. Tour Manage. (2015) 46:64–79. 10.1016/j.tourman.2014.06.006

[20] Shi S Wang Y Chen X Zhang Q. Conceptualization of omnichannel customer experience and its impact on shopping intention: a mixed-method approach. Int J Inf Manage. (2020) 50:325–36. 10.1016/j.ijinfomgt.2019.09.001

[21] Shen X Li Y Sun Y Wang N. Channel integration quality, perceived fluency and omnichannel service usage: the moderating roles of internal and external usage experience. Decis Support Syst. (2018) 109:61–73. 10.1016/j.dss.2018.01.006

[22] Rogers EM. Diffusion of innovations: Modifications of a model for telecommunications. In: Stoetzer MW Mahler A editors. Die Diffusion von Innovationen in der Telekommunikation. Schriftenreihe des Wissenschaftlichen Instituts für Kommunikationsdienste, Vol 17. Berlin: Springer (1995). 10.1007/978-3-642-79868-9_2

[23] Lou H Luo W Strong D. Perceived critical mass effect on groupware acceptance. Eur J Inform Syst. (2000) 9:91–103. 10.1057/palgrave.ejis.3000358

[24] Van Slyke C Ilie V Lou H Stafford T. Perceived critical mass and the adoption of a communication technology. Eur J Inform Syst. (2007) 16:270–83. 10.1057/palgrave.ejis.3000680

[25] Strader TJ Ramaswami SN Houle PA. Perceived network externalities and communication technology acceptance. Eur J Inform Syst. (2007) 16:54–65. 10.1057/palgrave.ejis.3000657

[26] Pae JH Hyun JS. The impact of technology advancement strategies on consumers' patronage decisions. J Prod Innov Manage. (2002) 19:375–83. 10.1111/1540-5885.1950375

[27] Davis FD. A technology acceptance model for empirically testing new end-user information systems: theory and results (PhD Dissertation). Massachusetts Institute of Technology, Sloan School of Management, Cambridge, MA, United States (1986).

[28] Sreelakshmi CC Prathap SK. Continuance adoption of mobile-based payments in COVID-19 context: An integrated framework of health belief model and expectation confirmation model. Int J Pervasive Comput Commun. (2020) 16:351–69. 10.1108/IJPCC-06-2020-0069

[29] Leung L Chen C. E-Health/M-health adoption and lifestyle improvements: exploring the roles of technology readiness, the expectation-confirmation model, and health-related information activities. Telecomm Policy. (2019) 43:563–75. 10.1016/j.telpol.2019.01.005

[30] Kim J Park H. Development of a health information technology acceptance model using consumers' health behavior intention. J Med Internet Res. (2012) 14:e133. 10.2196/jmir.2143 23026508 PMC3510715

[31] Ambalov IA. A meta-analysis of IT continuance: An evaluation of the expectation-confirmation model. Telematics Inform. (2018) 35:1561–71. 10.1016/j.tele.2018.03.016

[32] Alam M Parveen R Khan IR. Role of information technology in Covid-19 prevention. int J Bus Manag stud. (2020) 5:65–78.

[33] Sharma SK Sharma M. Examining the role of trust and quality dimensions in the actual usage of mobile banking services: An empirical investigation. Int J Inf Manage. (2019) 44:65–75. 10.1016/j.ijinfomgt.2018.09.013

[34] Alalwan AA Rana NP Dwivedi YK Algharabat R. Social media in marketing: a review and analysis of the existing literature. Telemat Inform. (2017) 34:1177–90. 10.1016/j.tele.2017.05.008

[35] Boontarig W Chutimaskul W Chongsuphajaisiddhi V Papasratorn B. Factors influencing the Thai elderly intention to use smartphone for e-Health services. In: 2012 IEEE Symposium on Humanities, Science and Engineering Research. IEEE (2012). p. 479–83. 10.1109/SHUSER.2012.6268881

[36] Shani G Gunawardana A. Evaluating recommendation systems. In: Ricci F Rokach L Shapira B Kantor P editors. Recommender Systems Handbook. Boston, MA: Springer (2011). 10.1007/978-0-387-85820-3_8

[37] Mayer RC Davis JH Schoorman FD. An integrative model of organizational trust. Acad Manage Rev. (1995) 20:709–34. 10.2307/258792

[38] Burda D Teuteberg F. The role of trust and risk perceptions in cloud archiving — Results from an empirical study. J High Technol Manage Res. (2014) 25:172–87. 10.1016/j.hitech.2014.07.008

[39] Jarvenpaa SL Tractinsky N Vitale M. Consumer trust in an Internet store. Inform Technol Manage. (2000) 1:45–71. 10.1023/A:1019104520776

[40] Raschke RL Krishen AS Kachroo P. Understanding the components of information privacy threats for location-based services. J Inform Syst. (2014) 28:227–42. 10.2308/isys-50696

[41] Davis FD. Perceived usefulness, perceived ease of use, and user acceptance of information technology. MIS Q. (1989) 13:319. 10.2307/249008

[42] Venkatesh V Thong JY Xu X. Consumer acceptance and use of information technology: Extending the unified theory of acceptance and use of technology. MIS Q. (2012) 36:157. 10.2307/41410412

[43] Özkan S Bindusara G Hackney R. Facilitating the adoption of E-paymEnt systems: theoretical constructs and empirical analysis. J Enterp Inform Manage. (2010) 23:305–25. 10.1108/17410391011036085

[44] Urumsah D. Factors influencing consumers to use e-services in indonesian airline companies. In: Quaddus M Woodside AG editors. E-Services Adoption: Processes by Firms in Developing Nations (Bingley: Emerald Group Publishing Limited), p. 5–254.

[45] Chang HS Lee SC Ji YG. Wearable device adoption model with TAM and TTF. Int J Mob Commun. (2016) 14:518. 10.1504/IJMC.2016.078726

[46] Natarajan T Balasubramanian SA Kasilingam DL. Understanding the intention to use mobile shopping applications and its influence on price sensitivity. J Retail Consum Serv. (2017) 37:8–22. 10.1016/j.jretconser.2017.02.010

[47] Leong KM Wongbusarakum S Ingram RJ Mawyer A Poe MR. Improving representation of human well-being and cultural importance in conceptualizing the west hawai'i ecosystem. Front Mar Sci. (2019) 6:231. 10.3389/fmars.2019.00231

[48] Chen C. Investigating structural relationships between service quality, perceived value, satisfaction, and behavioral intentions for air passengers: evidence from Taiwan. Transport Res Part A Policy Pract. (2008) 42:709–17. 10.1016/j.tra.2008.01.007

[49] Sweeney JC Soutar GN. Consumer perceived value: the development of a multiple item scale. J Retail. (2001) 77:203–20. 10.1016/S0022-4359(01)00041-0

[50] Rajaguru R. Role of value for money and service quality on behavioural intention: a study of full service and low cost airlines. J Air Trans Manage. (2016) 53:114–22. 10.1016/j.jairtraman.2016.02.008

[51] Shang D Wu W. Understanding mobile shopping consumers' continuance intention. Indust. Manag. Data Syst. (2017) 117:213–27. 10.1108/IMDS-02-2016-0052

[52] Faul F Erdfelder E Buchner A Lang A. Statistical power analyses using G^*^Power 3.1: tests for correlation and regression analyses. Behav Res Methods. (2009) 41:1149–60. 10.3758/BRM.41.4.1149 19897823

[53] Hair JF Jr Matthews LM Matthews RL Sarstedt M. PLS-SEM or CB-SEM: updated guidelines on which method to use. Int J Multivar Data Anal. (2017) 1:107. 10.1504/IJMDA.2017.087624

[54] Dutta-Bergman MJ. Primary sources of health information: comparisons in the domain of health attitudes, health cognitions, and health behaviors. Health Commun. (2004) 16:273–88. 10.1207/S15327027HC1603_1 15265751

[55] Mowen JC. The 3M model of motivation and personality: Theory and empirical applications to consumer behavior. Boston, MA: Springer (2000). 10.1007/978-1-4757-6708-7

[56] Li F Zhou D Chen Y Yu Y Gao N Peng J. The association between health beliefs and fall-related behaviors and its implication for fall intervention among Chinese elderly. Int J Environ Res Public Health. (2019) 16:4774. 10.3390/ijerph16234774 31795234 PMC6926647

[57] Tan GW-H Ooi K-B. Gender and age: Do they really moderate mobile Tourism shopping behavior? Telematics Inform. (2018) 35:1617–42. 10.1016/j.tele.2018.04.009

[58] Walker RH Craig-Lees M Hecker R Francis H. Technology-enabled service delivery. Int J Serv Ind Manage. (2002) 13:91–106. 10.1108/09564230210421173

[59] Kim S Bae J Jeon H. Continuous intention on accommodation apps: integrated value-based adoption and expectation–confirmation model analysis. Sustainability. (2019) 11:1578. 10.3390/su11061578

[60] Wang H Tao D Yu N Qu X. Understanding consumer acceptance of healthcare wearable devices: an integrated model of UTAUT and TTF. Int J Med Inform. (2020) 139:104156. 10.1016/j.ijmedinf.2020.104156 32387819

[61] Aksoy NC Alan AK Kabadayi ET Aksoy A. Individuals' intention to use sports wearables: the moderating role of technophobia. Int J Sports Mark Sponsor. (2020) 21:225–45. 10.1108/IJSMS-08-2019-0083

[62] Kock N. Common method bias in PLS-SEM: A full collinearity assessment approach. Int J eCollab. (2015) 11:1–10. 10.4018/ijec.2015100101

[63] Hair JF Ringle CM Sarstedt M. PLS-SEM: indeed a silver bullet. J Mark Theory Pract. (2011) 19:139–52. 10.2753/MTP1069-6679190202

[64] Podsakoff PM MacKenzie SB Podsakoff NP. Sources of method bias in social science research and recommendations on how to control it. Annu Rev Psychol. (2012) 63:539–69. 10.1146/annurev-psych-120710-100452 21838546

[65] Al Mamun A Fazal SA. Effect of entrepreneurial orientation on competency and micro-enterprise performance. Asia Pacific J Innovat Entrep. (2018) 12:379–98. 10.1108/APJIE-05-2018-0033

[66] Hair JF Jr Hult GTM Ringle C Sarstedt M. A Primer on Partial Least Squares Structural Equation Modeling (PLS-SEM). 2nd ed. Thousand Oaks, CA: Sage (2016).

[67] Korkmaz Ö Çakir R Özden MY. A validity and reliability study of the computational thinking scales (CTS). Comput Human Behav. (2017) 72:558–69. 10.1016/j.chb.2017.01.005

[68] Dijkstra TK Henseler J. Consistent partial least squares path modeling. MIS Q. (2015) 39:297–316. 10.25300/MISQ/2015/39.2.0229515491

[69] Hair JF Hult GT Ringle CM Sarstedt M Danks NP Ray S. Partial least squares structural equation modeling (PLS-SEM) using R. New York, NY; Cham: Springer (2021). 10.1007/978-3-030-80519-7

[70] Fornell C Larcker DF. Structural equation models with unobservable variables and measurement error: algebra and statistics. J Mark Res. (1981) 18:382–8. 10.1177/002224378101800313

[71] Kenny DA. Reflections on the actor-partner interdependence model. Pers Relation. (2018) 25:160–70. 10.1111/pere.12240

[72] Hayat N Al Mamun A Azwin N Nawi NC. Predictive accuracy comparison between structural equation modelling and neural network approach: A case of intention to adopt conservative agriculture practices. In: Alareeni B Hamdan A Elgedawy I editors. The Importance of New Technologies and Entrepreneurship in Business Development: In The Context of Economic Diversity in Developing Countries. Cham: Springer (2021). 10.1007/978-3-030-69221-6_141

[73] Goodrich K Benden M Munch J Wamwara W. Will college students take a stand? Effects of health orientations on purchase decision factors for standing desks. J Prod Brand Manage. (2020) 30:949–63. 10.1108/JPBM-07-2019-2481

[74] Nguyen DV Ross V Vu AT Brijs T Wets G Brijs K. Exploring psychological factors of mobile phone use while riding among motorcyclists in Vietnam. Transp Res Part F Traffic Psychol Behav. (2020) 73:292–306. 10.1016/j.trf.2020.06.023

[75] Chrysochou P Grunert KG. Health-related ad information and health motivation effects on product evaluations. J Bus Res. (2014) 67:1209–17. 10.1016/j.jbusres.2013.05.001

[76] Trakulmaykee N Benrit P. Investigating determinants and interaction quality effects on tourists' intention to use mobile tourism guide. Int J Innovat Technol Manage. (2015) 12:1550005. 10.1142/S0219877015500054

[77] Truong TH. The drivers of omni-channel shopping intention: a case study for fashion retailing sector in Danang, Vietnam. J Asian Bus Econ Stud. (2020) 28:143–59. 10.1108/JABES-05-2020-0053

[78] Zhang H Lu Y Gupta S Gao P. Understanding group-buying websites continuance. Internet Res. (2015) 25:767–93. 10.1108/IntR-05-2014-0127

[79] Wu K Vassileva J Zhao Y. Understanding users' intention to switch personal cloud storage services: evidence from the Chinese market. Comput Human Behav. (2017) 68:300–14. 10.1016/j.chb.2016.11.039

[80] Cheng F Wu C Chen Y. Creating customer loyalty in online brand communities. Comput Human Behav. (2020) 107:105752. 10.1016/j.chb.2018.10.018

[81] Liu F Ngai E Ju X. Understanding mobile health service use: an investigation of routine and emergency use intentions. Int J Inf Manage. (2019) 45:107–17. 10.1016/j.ijinfomgt.2018.09.004

[82] Park E Kim KJ Kwon SJ. Understanding the emergence of wearable devices as next-generation tools for health communication. Inform Technol People. (2016) 29:717–32. 10.1108/ITP-04-2015-0096

[83] Zhao Y Ni Q Zhou R. What factors influence the mobile health service adoption? A meta-analysis and the moderating role of age. Int J Inform Manage. (2018) 43:342–50. 10.1016/j.ijinfomgt.2017.08.006

[84] Gunawardana GH Kulathunga D Perera W. Impact of self service technology quality on customer satisfaction: a case of retail banks in western province in Sri Lanka. Gadjah Mada Int J Bus. (2015) 17:1. 10.22146/gamaijb.6147

[85] Riskinanto A Kelana B Hilmawan DR. The moderation effect of age on adopting E-payment technology. Procedia Comput Sci. (2017) 124:536–43. 10.1016/j.procs.2017.12.187

[86] Dahab D Bouqlila F. Factors influencing e-payment adoption case study: Pandemic COVID-19. In: Al-Emran M Al-Sharafi MA Al-Kabi MN Shaalan K editors. Proceedings of International Conference on Emerging Technologies and Intelligent Systems. Cham: Springer (2022). 10.1007/978-3-030-85990-9_53

[87] Al-Saedi K Al-Emran M Ramayah T Abusham E. Developing a general extended UTAUT model for M-payment adoption. Technol Soc. (2020) 62:101293. 10.1016/j.techsoc.2020.101293

[88] Dwivedi YK Rana NP Tamilmani K Raman R. A meta-analysis based modified unified theory of acceptance and use of technology (meta-UTAUT): a review of emerging literature. Curr Opin Psychol. (2020) 36:13–8. 10.1016/j.copsyc.2020.03.008 32339928

[89] Im I Hong S Kang MS. An international comparison of technology adoption. Inform Manage. (2011) 48:1–8. 10.1016/j.im.2010.09.001

[90] Ahmad SZ Abu Bakar AR Faziharudean TM Mohamad Zaki KA. An empirical study of factors affecting e-Commerce adoption among small- and medium-sized enterprises in a developing country: evidence from Malaysia. Inform Technol Dev. (2014) 21:555–72. 10.1080/02681102.2014.899961

[91] Islam AN. E-learning system use and its outcomes: moderating role of perceived compatibility. Telemat Inform. (2016) 33:48–55. 10.1016/j.tele.2015.06.010

[92] Wang Y Li H Li C Zhang D. Factors affecting hotels' adoption of mobile reservation systems: a technology-organization-environment framework. Tour Manage. (2016) 53:163–72. 10.1016/j.tourman.2015.09.021

[93] Alalwan AA Dwivedi YK Rana NP Simintiras AC. Jordanian consumers' adoption of telebanking. Int J Bank Mark. (2016) 34:690–709. 10.1108/IJBM-06-2015-0093

[94] Zhang M Luo M Nie R Zhang Y. Technical attributes, health attribute, consumer attributes and their roles in adoption intention of healthcare wearable technology. Int J Med Inform. (2017) 108:97–109. 10.1016/j.ijmedinf.2017.09.016 29132639

[95] Pentina I Zhang L Bata H Chen Y. Exploring privacy paradox in information-sensitive mobile app adoption: a cross-cultural comparison. Comput Human Behav. (2016) 65:409–19. 10.1016/j.chb.2016.09.005

[96] Hsu C Lin JC. What drives purchase intention for paid mobile apps? – An expectation confirmation model with perceived value. Electron Commerce Res Appl. (2015) 14:46–57. 10.1016/j.elerap.2014.11.003

[97] Karjaluoto H Shaikh AA Saarijärvi H Saraniemi S. How perceived value drives the use of mobile financial services apps. Int J Inf Manage. (2019) 47:252–61. 10.1016/j.ijinfomgt.2018.08.014

[98] Sullivan YW Kim DJ. Assessing the effects of consumers' product evaluations and trust on repurchase intention in e-Commerce environments. Int J Inf Manage. (2018) 39:199–219. 10.1016/j.ijinfomgt.2017.12.008

[99] Dwivedi YK Rana NP Jeyaraj A Clement M Williams MD. Re-examining the unified theory of acceptance and use of technology (UTAUT): towards a revised theoretical model. Inform Syst Front. (2019) 21:719–34. 10.1007/s10796-017-9774-y

[100] Shin G Jarrahi MH Fei Y Karami A Gafinowitz N Byun A. Wearable activity trackers, accuracy, adoption, acceptance and health impact: a systematic literature review. J Biomed Inform. (2019) 93:103153. 10.1016/j.jbi.2019.103153 30910623

[101] Mosteller J Poddar A. To share and protect: using regulatory focus theory to examine the privacy paradox of consumers' social media engagement and online privacy protection behaviors. J Interact Mark. (2017) 39:27–38. 10.1016/j.intmar.2017.02.003

[102] Jia B Zhou T Li W Liu Z Zhang J. A blockchain-based location privacy protection incentive mechanism in crowd sensing networks. Sensors. (2018) 18:3894. 10.3390/s18113894 30424534 PMC6263764

[103] Hilty DM Maheu MM Drude KP Hertlein KM Wall K Long RP. Telebehavioral health, telemental health, E-ThErapy and e-Health competencies: the need for an interprofessional framework. J Technol Behav Sci. (2017) 2:171–89. 10.1007/s41347-017-0036-0

